# Effectiveness of PARP inhibition in enhancing the radiosensitivity of 3D spheroids of head and neck squamous cell carcinoma

**DOI:** 10.3389/fonc.2022.940377

**Published:** 2022-08-16

**Authors:** Chumin Zhou, Maria Rita Fabbrizi, Jonathan R. Hughes, Gabrielle J. Grundy, Jason L. Parsons

**Affiliations:** ^1^ Department of Molecular and Clinical Cancer Medicine, University of Liverpool, Liverpool, United Kingdom; ^2^ Clatterbridge Cancer Centre NHS Foundation Trust, Bebington, United Kingdom

**Keywords:** DNA repair, head and neck cancer, ionizing radiation, PARP, proton beam therapy, HNSCC, HPV, radiosensitisation

## Abstract

A critical risk factor for head and neck squamous cell carcinoma (HNSCC), particularly of the oropharynx, and the response to radiotherapy is human papillomavirus (HPV) type-16/18 infection. Specifically, HPV-positive HNSCC display increased radiosensitivity and improved outcomes, which has been linked with defective signalling and repair of DNA double-strand breaks (DSBs). This differential response to radiotherapy has been recapitulated *in vitro* using cell lines, although studies utilising appropriate 3D models that are more reflective of the original tumour are scarce. Furthermore, strategies to enhance the sensitivity of relatively radioresistant HPV-negative HNSCC to radiotherapy are still required. We have analysed the comparative response of *in vitro* 3D spheroid models of oropharyngeal squamous cell carcinoma to x-ray (photon) irradiation and provide further evidence that HPV-positive cells, in this case now grown as spheroids, show greater inherent radiosensitivity compared to HPV-negative spheroids due to defective DSB repair. We subsequently analysed these and an expanded number of spheroid models, with a particular focus on relatively radioresistant HPV-negative HNSCC, for impact of poly(ADP-ribose) polymerase (PARP) inhibitors (olaparib and talazoparib) in significantly inhibiting spheroid growth in response to photons but also proton beam therapy. We demonstrate that in general, PARP inhibition can further radiosensitise particularly HPV-negative HNSCC spheroids to photons and protons leading to significant growth suppression. The degree of enhanced radiosensitivity was observed to be dependent on the model and on the tumour site (oropharynx, larynx, salivary gland, or hypopharynx) from which the cells were derived. We also provide evidence suggesting that PARP inhibitor effectiveness relates to homologous recombination repair proficiency. Interestingly though, we observed significantly enhanced effectiveness of talazoparib versus olaparib specifically in response to proton irradiation. Nevertheless, our data generally support that PARP inhibition in combination with radiotherapy (photons and protons) should be considered further as an effective treatment for HNSCC, particularly for relatively radioresistant HPV-negative tumours.

## Introduction

A worldwide incidence of ~800,000 cases each year of head and neck squamous cell carcinoma (HNSCC) has been reported (1), with regional and local recurrence plus distant metastasis predominantly causing ~60% of the mortality rates. The major risk factors of this disease comprise excessive alcohol consumption, smoking, and human papillomavirus (HPV) type-16/18 infection, the latter of which accounts for ~60% of oropharyngeal squamous cell carcinoma (OPSCC) (2–4). Furthermore, HPV-positive OPSCC patients display a better clinical prognosis and survival rates compared to HPV-negative OPSCC through an enhanced response to radiotherapy and chemotherapy (5–8). Recent *in vitro* studies have recapitulated the enhanced radiosensitivity of HPV-positive OPSCC cell lines grown as monolayers in comparison to the respective HPV-negative cell models (9–12). Furthermore, and given that the therapeutic effect of radiotherapy (ionising radiation; IR) is achieved through the generation of DNA damage, there is collective evidence in these and other studies to suggest that the inherent increased radiosensitivity of HPV-positive OPSCC is caused by defects in the cellular DNA damage response (DDR) (13). Specifically, it has been shown that there is delayed repair of DNA double-strand breaks (DSBs), measured directly but also using surrogate markers such as γH2AX and 53BP1, in response to photon irradiation in HPV-positive OPSCC cells. The precise impact of HPV infection on DSB repair proficiency is still unclear though, as both reduced expression and activities of enzymes involved in both homologous recombination (HR) and non-homologous end joining (NHEJ), the two major DNA DSB repair pathways, have been shown (9, 10). Nevertheless, it is apparent that the DDR plays a critical role in determining the radiosensitivity of HNSCC cell lines *in vitro*. Importantly however, the utilisation of 3D models of HNSCC (such as spheroids and patient-derived organoids) that more accurately reflect the structure and environment of the original tumour and their response to IR mediated *via* the DDR is less well known.

Poly (ADP-ribose) polymerases (PARPs) are a family of 17 enzymes that predominantly play an essential role in post-translational modification of target proteins through attachment of ADP-ribose units using NAD+ as a substrate (14). Only three PARPs (specifically PARP1, PARP2, and PARP3) are mainly engaged in the DDR, where they play immediate roles in DNA strand break binding and aid in the processes of base excision repair (BER) and DSB repair by HR and NHEJ (15). PARP inhibition has proven to be an effective strategy for the killing of BRCA-deficient tumour cells through a process known as synthetic lethality (16, 17). This takes advantage of the inability of these cells to process DSBs through HR, and through the action of inhibiting PARPs involved in the repair of DNA single-strand breaks, this leads to accumulation of replication-induced and toxic DSBs. An increasing number of studies have suggested that PARP inhibition, using predominantly either veliparib or olaparib, leads to the accumulation of DSBs and enhanced radiosensitivity of both HPV-positive and HPV-negative HNSCC cells [reviewed in 18)]. However, there is conflicting evidence to suggest whether DSB repair-defective HPV-positive HNSCC cells are more effectively sensitised by PARP inhibition to IR. Also comparatively, the sensitivity of relatively radioresistant HPV-negative HNSCC cells appears largely responsive to PARP inhibitors even though these are deemed DSB repair proficient. A notable point is that the effectiveness of radiosensitisation by PARP inhibitors may relate to their catalytic inhibition (IC_50_), PARP trapping potency (retaining PARP protein on the DNA strand break site), or the combination of both (19, 20). To this effect, it is known that veliparib is a relatively weak PARP trapper whereas increasing trapping ability is observed with olaparib, but more so talazoparib is deemed a strong PARP trapper (21, 22). However, the comparative ability of different PARP inhibitors to radiosensitise HNSCC cells and 3D spheroid models has not been studied in detail.

In this study, we have developed 3D spheroid models of HPV-positive and HPV-negative OPSCC and analysed their growth in response to x-rays (photons) but also proton irradiation. We demonstrate that HPV-positive OPSCC grown as 3D spheroids are more radiosensitive, compared with HPV-negative OPSCC spheroids and that this correlates with slower rates of DSB repair. Subsequently, we show that radiosensitivity of OPSCC spheroids can be increased by PARP inhibition (olaparib and talazoparib), particularly within a larger number of relatively radioresistant HPV-negative HNSCC spheroids, and that this is evident in response to both x-rays and protons. Given that 3D spheroid models act as more representative models of the original patient tumour, this research suggests that PARP inhibition in combination with radiotherapy should be investigated further as an effective combinatorial treatment for HNSCC and particularly for HPV-negative disease.

## Methods and materials

### Cell lines and culture conditions

HPV-positive OPSCC cells (UPCI-SCC090 and UPCI-SCC154) were kindly provided by Dr. S. Gollin, University of Pittsburgh. HPV-negative OPSCC cells (UMSCC6, UMSCC74A) and those from the larynx (UMSCC11B, UMSCC17A) were kindly provided by Prof. T. Carey, University of Michigan, USA. HPV-negative HNSCC cells from the salivary gland (A253) and hypopharynx (Detroit 562, FaDu) originated from ATCC (Teddington, UK). All cells, apart from UPCI-SCC090, UPCI-SCC154, Detroit 562, and FaDu [which were cultured in Minimal Essential Medium (MEM)], were routinely cultured as monolayers in Dulbecco’s Modified Eagle Medium (DMEM) with 10% foetal bovine serum, 1× non-essential amino acid, 2 mM L-glutamine, and 1× penicillin–streptomycin. All cell lines were maintained and incubated in 5% CO_2_ at 37°C and were authenticated in our laboratory by STR profiling.

### Spheroid growth assay

Cells were seeded at 500–1,000 cells/well in triplicate in 100 µl Advanced MEM (Life Technologies, Paisley, UK) containing 1% B27 supplement, 0.5% N2 supplement, 2 mM L-glutamine, 1× penicillin–streptomycin, 5 µg/ml heparin, 20 ng/µl epithermal growth factor (EGF), and 10 ng/µl fibroblast growth factor (FGF) in 96-well ultra-low attachment plates (Corning B.V. Life Sciences, Amsterdam, The Netherlands). After 24 h, the PARP inhibitors olaparib (AZD2281; Selleckchem, Munich, Germany) and talazoparib (BMN673; AbMole BioScience, Brussels, Belgium) were added to a concentration of 0.1 µM to the spheroids. After another 24 h at which the spheroids were ~200 µm in size, they were subsequently irradiated using a CellRad x-ray irradiator (Faxitron Bioptics, Tucson, USA) at a dose rate of ~3 Gy/min, or alternatively with a passive scattered horizontal proton beam line of 60 MeV maximal energy at a dose rate of ~5 Gy/min as previously described (23, 24). Higher doses of protons versus photons were comparatively used due to positioning of spheroids at the entrance dose of a pristine (unmodulated) beam (~1 keV/µm). Immediately following irradiation, 50 µl culture media was removed and replaced by 50 µl fresh media (without inhibitor). The growth of spheroids was monitored up to 15 days post-seeding by image capture using the EVOS M5000 Imaging System (Life Technologies, Paisley, UK). The diameter (d) of the spheroids was measured by using ImageJ which was converted into spheroid volume (V) by using the formula V = 4/3 × π(d/2)^3^.

### Spheroid neutral comet assays

Spheroids were irradiated 48 h post-seeding with 4 Gy x-rays and harvested at various time points (0–240 min) post-IR. Spheroids (~10 per time point) were collected and centrifuged (1,000 × *g* for 10 min at 4°C), the supernatant was removed, and spheroids were washed with PBS. Spheroids were re-centrifuged and resuspended in 1× trypsin-EDTA for ~2 min at 37°C until single cells were generated, and diluted to ~1 × 10^5^ cells/ml using cell culture media. The neutral comet assay was then used for measurement of the levels of DSBs, similar to that previously described (9). In brief, the cell suspension (20 µl) was mixed with 80 µl 1% low melting point agarose (Bio-Rad, Hemel Hempstead, UK) in PBS (molten and kept at 35°C) and embedded on a microscope slide precoated with 1% normal melting point agarose that had allowed to dry overnight. A 22 × 22 mm coverslip was added and the slide placed on ice to allow the agarose to set. Cell lysis was then performed by removing the coverslips and adding the slides to staining jars containing fresh cold lysis buffer (2.5 M NaCl, 100 mM EDTA disodium salt, 10 mM Tris base, 1% N-lauroylsarcosine, 1% DMSO, and 1% (v/v) Triton X-100; pH 9.5) and kept for at least 1 h at 4°C. Slides were then transferred to a dark comet assay tank (Appleton Woods, Birmingham, UK) and covered with fresh cold electrophoresis buffer containing 1× TBE (90 mM Tris–borate, 2 mM EDTA, pH 8.3) to allow the DNA to unwind. Electrophoresis was then performed at 25 V, ~15 mA for 25 min. Slides were removed from the comet assay tank and washed three times with 1× PBS (5 min each each) before being allowed to air dry overnight. Slides were rehydrated in dH_2_O (pH 8.0) for 30 min, the DNA was stained with SYBR Gold (Life Technologies, Paisley, UK) diluted 1:20,000 in dH_2_O (pH 8.0) for 30 min, and then slides were left to air dry again overnight. Comets were visualised using an Olympus fluorescent microscope with a Photometrics CoolSNAP HQ2 CCD camera, and images were captured using Micro-Manager Software. Images of comets were analysed using Komet 6.0 image analysis software (Andor Technology, Belfast, Northern Ireland) to determine % tail DNA values. Experimental data were collected from at least three independent, biological experiments.

### Immunoblotting and immunofluorescent staining

Whole cell extracts were prepared from HNSCC cells and analysed by immunoblotting as previously described (9). RAD51 antibodies were from Bethyl Laboratories (Montgomery, USA), ATR antibodies were from Abcam (Cambridge, UK), CHK1 antibodies were from Cell Signalling Technology (Leiden, The Netherlands), and actin antibodies were from Merck-Sigma (Gillingham, UK). For immunofluorescent staining of RAD51, cells were grown on 13-mm coverslips, unirradiated or irradiated with 4 Gy x-rays, and allowed to repair for 4 h in 5% CO_2_ at 37°C, prior to fixing and staining as previously described (9).

### Statistical analysis

All experiments were performed in at least triplicate as separate independent, biological experiments and expressed as mean ± standard deviations. Changes in growth of spheroids post-irradiation, in the absence or presence of PARP inhibition, were analysed by determining the fold increase in spheroid volume between days 3 and 11 (protons) or 12 (x-rays) post-seeding in the DMSO control, versus the fold increases following treatment. Statistical analysis of DSBs quantified through neutral comet assays, and RAD51 foci through immunofluorescent staining, was performed using a one-sample *t*-test.

## Results

### HPV-positive are more radiosensitive than HPV-negative OPSCC spheroids to x-ray radiation

We have previously demonstrated that the radiosensitivity of HPV-positive OPSCC cells grown as monolayers is higher than the corresponding HPV-negative cells, largely due to the defective efficiency in repair of DNA DSBs post-IR (9). This has been replicated in other studies (10, 11). To examine if this phenotype is recapitulated in 3D spheroid models, we used three of the four same OPSCC cell lines used in our previous study and where the expression of p16 as a marker of E6 and E7 oncogenes in HPV-positive cells was confirmed (note that UMSCC47 cells, which routinely did not form or grow spheroids, were replaced with UPCI-SCC154). Our initial observations were that the spheroids from the HPV-negative cells (UMSCC6 and UMSCC74A) grew linearly up to 10–12 days post-seeding, where they increased in volume by 9.4–12.2-fold, and growth subsequently ceased from day 12 onwards (**Supplementary Figures 1A**, **B**). In response to a single dose of x-ray (photon) irradiation, the growth of the HPV-negative OPSCC spheroids was reduced by 30%–46% at 1 Gy, 45%–60% at 2 Gy, and there was limited spheroid growth following a dose of 5 Gy. In contrast, the spheroids from the HPV-positive cells (UPCI-SCC090 and UPCI-SCC154) displayed different growth characteristics. UPCI-SCC090-derived spheroids had delayed growth but which started to increase linearly from day 8 post-seeding onwards and reached an 11-fold increase in volume by day 15 (**Supplementary Figure 1C**). However, UPCI-SCC154-derived spheroids only grew by ~1.6-fold in volume at 10 to 15 days post-seeding (**Supplementary Figure 1D**). Despite these differential growth kinetics in comparison to HPV-negative OPSCC spheroids, HPV-positive OPSCC spheroid growth was significantly inhibited by a single 1 Gy dose of x-rays and completely inhibited by either a 2 or 5 Gy dose (**Supplementary Figures 1C, D**).

**Figure 1:**
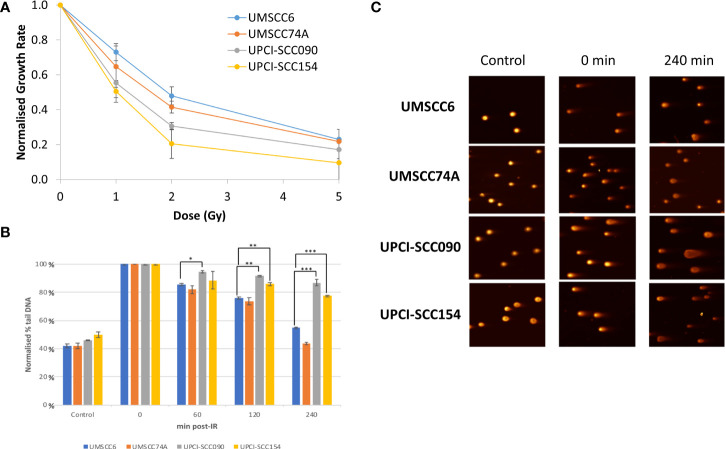
Analysis of the efficiency of repair of IR-induced DSBs in HPV-positive and HPV-negative OPSCC spheroids. **(A)** Spheroids were allowed to develop for 48 h in ultra-low attachment plates and then unirradiated or irradiated (1, 2, or 5 Gy) on day 3 with a single dose of x-rays. The rate in growth of HPV-negative OPSCC spheroids (UMSCC6 and UMSCC74A) and HPV-positive OPSCC spheroids (UPCI-SCC090 and UPCI-SCC154) measured by microscopy from day 3 to day 12 was calculated following each dose of radiation and normalised against the unirradiated controls (set to 1.0). Data were analysed from three biologically independent experiments. **(B, C)** Spheroids were allowed to develop for 48 h in ultra-low attachment plates and then unirradiated or irradiated (5 Gy) with a single dose of x-rays. Spheroids were harvested at the relevant time points post-irradiation (0-240 min), trypsinised into single cells and DSB levels measured using the neutral comet assay. **(B)** Shown is the mean % tail DNA with standard deviations from three independent biological experiments, normalised to the DNA DSBs levels at 0 min post-IR, which was set to 100%. *p < 0.02, **p < 0.01, ***p < 0.005 as analysed by a one sample t-test. **(C)** Representative images of comets derived from OPSCC spheroids acquired from unirradiated controls and immediately or 240 min post-IR.

In order to directly compare the radiosensitivity of HPV-negative (UMSCC6 and UMSCC74A) and HPV-positive (UPCISCC090 and UPCISCC154) OPSCC spheroids, the rate in growth of spheroid volume between days 3 and 12 (when all spheroid models were still actively growing) was calculated following each dose of photon radiation and normalised against the unirradiated controls (set to 1.0). This demonstrated that the spheroid radiosensitivity, as a function of growth, was generally in the order UMSCC6 > UMSCC74A > UPCISCC090 > UPCISCC154 (**Figure 1A**). These data are very similar to that which we previously acquired using clonogenic survival assays (9) but which further show that HPV-negative OPSCC cells grown as 3D spheroids are comparatively more radioresistant than those from HPV-positive cells. In addition to measuring spheroid growth, we analysed the DSB repair efficiency of OPSCC cells grown as 3D spheroids following photon irradiation. Spheroids from each cell line were harvested at 0–240 min post-irradiation, disrupted using trypsin, and the single cells thus generated were processed using neutral comet assays to quantify the levels and repair of DSB damage (note the ~12 min sample processing time at 4°C which should be taken into account in regard to these stated analysis times). Following normalisation of the data immediately post-irradiation (set to 100%), it was observed that DSB levels (expressed as % tail DNA) of cells from HPV-negative OPSCC spheroids (UMSCC6 and UMSCC74A) gradually reduced over the 240 min time period at which point the levels were similar to those in the unirradiated control (**Figures 1B, C**). It should be noted that the DSB levels in the control (unirradiated) samples were relatively high (~40% tail DNA) due to the action of the trypsin required to effectively disrupt the spheroids into single cells, but also that these are relative to those in the irradiated samples after data normalisation. In contrast, we observed in cells from HPV-positive OPSCC spheroids (UPCI-SCC090 and UPCI-SCC154) that the levels of DSBs still remained high at 120 and 240 min post-irradiation and were significantly different from DSB levels in cells from HPV-negative OPSCC spheroids (UMSCC6 and UMSCC74A) (**Figures 1B, C**). This demonstrates reduced repair efficiency of IR-induced DSBs in the HPV-positive OPSCC spheroids compared with their HPV-negative counterparts, which reproduces previously shown evidence using monolayer cells.

### Olaparib enhances the radiosensitivity of selective HPV-negative HNSCC spheroids

**Figure 2:**
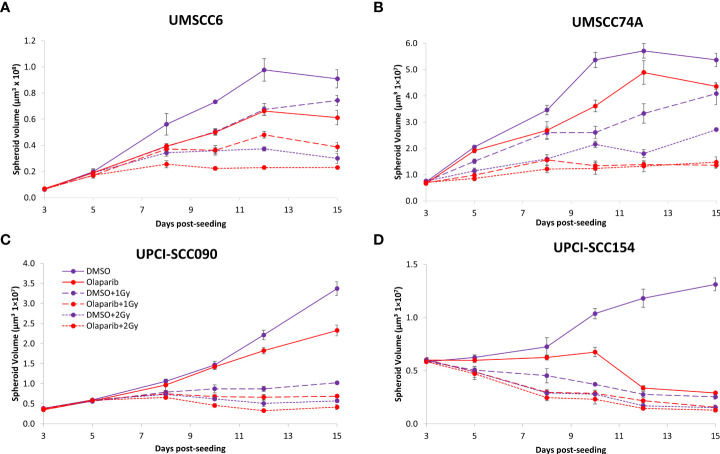
Impact of olaparib on the radiosensitivity and growth of HPV-negative and HPV-positive OPSCC spheroids. Spheroids were allowed to develop for 24 h in ultra-low-attachment plates, treated with DMSO or olaparib (0.1 µM) for a further 24 h, and then unirradiated or irradiated (1 or 2 Gy) on day 3 with a single dose of x-rays. Growth of **(A, B)** HPV-negative OPSCC spheroids (UMSCC6 and UMSCC74A) and **(C, D)** HPV-positive OPSCC spheroids (UPCI-SCC090 and UPCI-SCC154) was measured by microscopy up to 15 days post-seeding and analysed from three biologically independent experiments.

**Figure 3:**
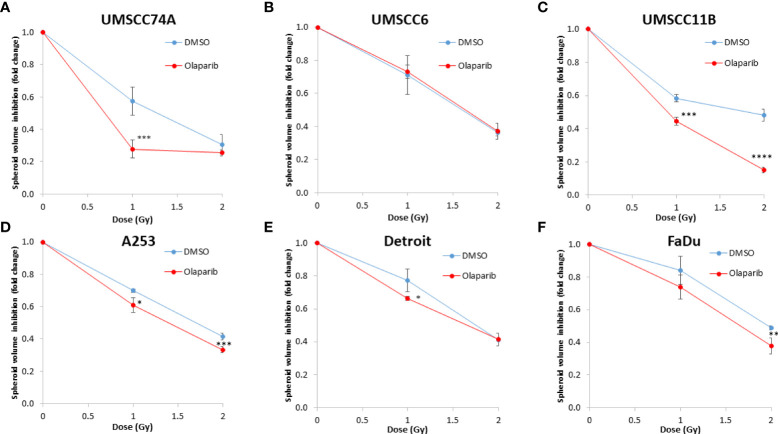
Impact of olaparib on the enhancement of the radiosensitivity of HPV-negative HNSCC spheroids. **(A–F)** The fold growth of HPV-negative HNSCC spheroids from days 3 to 12 post-seeding was determined relative to the x-ray radiation dose, and this was normalised to the unirradiated control which was set to 1.0. *p < 0.05, **p < 0.02, ***p < 0.01, ****p < 0.001 as analysed by a two-sample t-test.

We examined whether the radiosensitivity of both HPV-negative and HPV-positive OPSCC spheroids could be enhanced with the PARP inhibitor, olaparib. The inhibitor (0.1 µM) was added to the spheroids 24 h post-seeding, a concentration that was effective at supressing radiation-induced poly(ADP-ribosyl)ation (**Supplementary Figure 2A**). After 24 h of incubation, the spheroid was irradiated with a single dose of x-rays (1 or 2 Gy), and growth rates of all OPSCC spheroids were monitored up to 12–15 days post-seeding. We observed that olaparib alone was able to supress the growth of HPV-negative OPSCC 3D spheroids (UMSCC6 and UMSCC74A) by 1.1–1.6-fold (**Figures 2A, B**, **Table 1**, **Supplementary Figure 3**). However, in combination with irradiation, olaparib was also able to effectively supress growth by 1.5–2.2-fold (1 Gy) and by 1.3–1.6-fold (2 Gy) compared against the respective DMSO-treated spheroids. The data were further analysed by measuring the fold decrease in spheroid volume relative to the dose of radiation, as a demonstration of radiosensitivity enhancement through synergy with PARP inhibition. This revealed that only UMSCC74A spheroids were significantly radiosensitised in a synergistic manner particularly at a 1 Gy dose of x-rays in combination with olaparib, whereas there was no difference in enhanced radiosensitisation of UMSCC6 spheroids (**Figures 3A, B**). In terms of HPV-positive OPSCC spheroids, olaparib alone appeared to have an impact on inhibiting the growth of particularly the UPCI-SCC154 spheroids where a 3.6-fold reduction in growth was observed (**Figures 2C, D**, **Table 1**, **Supplementary Figure 3**), although in combination with irradiation, olaparib had a relatively reduced impact on HPV-positive OPSCC spheroid growth. This is evidenced by reductions in growth by only 1.3-fold (1 Gy) and by 1.1–1.5-fold (2 Gy). Overall, this demonstrates the inherent increased radiosensitivity of the HPV-positive OPSCC models. This is also despite the HPV-positive OPSCC cells containing comparatively higher protein levels of PARP-1 (**Supplementary Figure 2B**), which we have also observed previously (9).

**Table 1:** Olaparib enhances the sensitivity of HPV-negative OPSCC spheroids in response to x-ray irradiation.

Treatment	UMSCC6	UMSCC74A	UPCI-SCC090	UPCI-SCC154
Olaparib	1.6 ± 0.1	1.1 ± 0.1	1.1 ± 0.2	3.6 ± 0.5
Olaparib+1 Gy	1.5 ± 0.2	2.2 ± 0.3	1.3 ± 0.1	1.3 ± 0.1
Olaparib+2 Gy	1.6 ± 0.2	1.3 ± 0.2	1.5 ± 0.2	1.1 ± 0.0

Growth inhibition ratios (mean ± S.D) comparing the fold increase in spheroid volume between days 3 and 12 following olaparib versus the appropriate DMSO controls (alone, or combination with x-rays) were calculated in HPV-negative and HPV-positive OPSCC spheroids.

**Figure 4:**
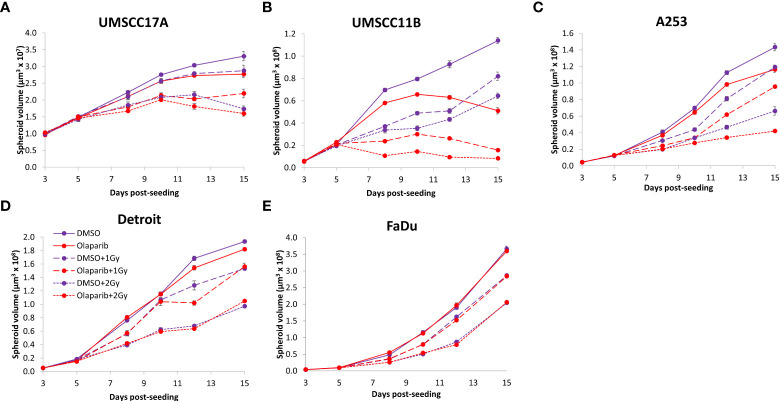
Impact of olaparib on the radiosensitivity and growth of HPV-negative HNSCC spheroids. Spheroids were allowed to develop for 24 h in ultra-low attachment plates, treated with DMSO or olaparib (0.1 µM) for a further 24 h, and then unirradiated or irradiated (1 or 2 Gy) on day 3 with a single dose of x-rays. Growth of spheroids derived from cells from **(A, B)** the larynx (UMSCC17A and UMSCC11B), **(C)** the salivary gland (A253), and **(D, E)** the hypopharynx (Detroit 562 and FaDu) were measured by microscopy up to 15 days post-seeding and analysed from three biologically independent experiments.

Given the known relative radioresistance of HPV-negative OPSCC cells and our observation that this is preserved in 3D spheroids, we extended our study by using spheroids grown from additional HPV-negative cell lines originating from the larynx (UMSCC11B and UMSCC17A), salivary gland (A253), and hypopharynx (Detroit 562 and FaDu) and examined their radiosensitivity in combination with olaparib. The two laryngeal spheroid models grew to different sizes over the 15-day period, either 3.3-fold (UMSCC17A) or 19.3-fold (UMSCC11B) (**Figures 4A, B**). Nevertheless, olaparib alone was able to supress the growth of laryngeal spheroids moderately by only 1.1–1.4-fold, but importantly olaparib enhanced the impact of x-ray irradiation in supressing growth of both UMSCC11B and UMSCC17A spheroids by 1.3–1.9-fold (1 Gy) and by 1.3–4.6-fold (2 Gy) compared against the respective DMSO-treated spheroids (**Figures 4A, B**, **Table 2**, **Supplementary Figure 4**). Using spheroids derived from cells of the salivary gland (A253), growth again was only moderately affected (1.1-fold) by olaparib alone, although this enhanced the response to irradiation (1.3–1.4-fold at 1 and 2 Gy) (**Figure 4C**, **Table 2**, **Supplementary Figure 4**). In contrast, spheroids derived from HPV-negative cells from the hypopharynx (Detroit 562 and FaDu) showed no sensitivity to olaparib only, and olaparib had a relatively minor impact on x-ray radiosensitivity (1.0–1.3-fold inhibition at 1 and 2 Gy) (**Figures 4D, E**, **Table 2**, **Supplementary Figure 4**). It was noticeable that both these hypopharyngeal cell lines contained comparatively lower PARP-1 protein levels that all of the others analysed (**Supplementary Figure 2B**). Interestingly, analysis of the TCGA database demonstrates that *parp1* mRNA expression is generally higher in HNSCC than normal tissues, but there is no statistical difference in expression across different HNSCC tumour sites (**Supplementary Figures 5A, B**). Nevertheless, analysis of fold decreases in spheroid volume relative to radiation dose to analyse for synergy with PARP inhibition further revealed significant radiosensitivity enhancement of UMSCC11B and A253 spheroids by olaparib, whereas there was only a mild impact of the treatment on FaDu (significant at 2 Gy dose only) and on Detroit 562 spheroids (significant at the 1 Gy dose only; **Figure 3C–F**).

**Table 2:** Olaparib and talazoparib selectively enhance the sensitivity of HPV-negative HNSCC spheroids in response to x-ray irradiation.

Treatment	UMSCC6	UMSCC74A	UMSCC17A	UMSCC11B	A253	Detroit 562	FaDu
Olaparib	1.6 ± 0.1	1.1 ± 0.1	1.1 ± 0.0	1.4 ± 0.1	1.1 ± 0.0	1.1 ± 0.1	0.9 ± 0.1
Ola+1 Gy	1.5 ± 0.2	2.2 ± 0.3	1.3 ± 0.1	1.9 ± 0.0	1.3 ± 0.0	1.3 ± 0.0	1.0 ± 0.1
Ola+2 Gy	1.6 ± 0.2	1.3 ± 0.2	1.3 ± 0.1	4.6 ± 0.7	1.4 ± 0.1	1.1 ± 0.0	1.2 ± 0.1
Talazoparib	2.2 ± 0.4	4.0 ± 0.6	n.d.	7.7 ± 1.0	1.7 ± 0.2	1.1 ± 0.3	1.3 ± 0.1
Tala+1 Gy	1.8 ± 0.1	n.d.	n.d.	6.6 ± 0.8	1.6 ± 0.4	1.2 ± 0.1	1.5 ± 0.2
Tala+2 Gy	2.4 ± 0.1	n.d.	n.d.	5.6 ± 0.4	2.0 ± 0.4	1.2 ± 0.3	2.7 ± 0.5

Growth inhibition ratios (mean ± S.D) comparing the fold increase in spheroid volume between days 3 and 12 following olaparib or talazoparib versus the appropriate DMSO controls (alone, or combination with x-rays) were calculated in HPV-negative and HPV-positive HNSCC spheroids. n.d. refers to not determined.

### Talazoparib additively enhances the radiosensitivity of HPV-negative HNSCC spheroids

**Figure 5:**
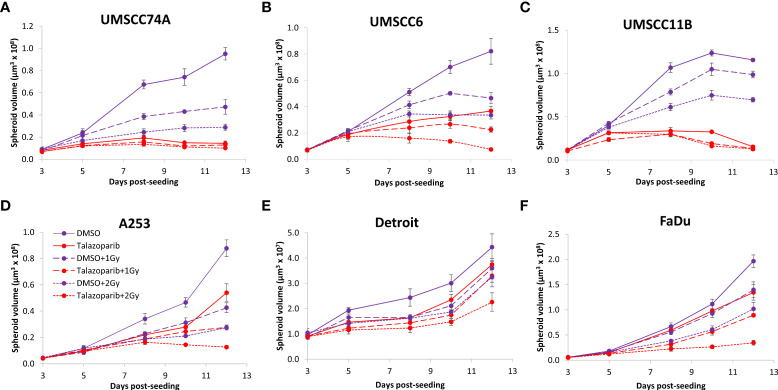
Impact of talazoparib on the radiosensitivity and growth of HPV-negative HNSCC spheroids. Spheroids were allowed to develop for 24 h in ultra-low attachment plates, treated with DMSO or talazoparib (0.1 µM) for a further 24 h, and then unirradiated or irradiated (1 or 2 Gy) on day 3 with a single dose of x-rays. Growth of spheroids derived from cells from **(A, B)** the oropharynx (UMSCC74A and UMSCC6), **(C)** the larynx (UMSCC11B), **(D)** the salivary gland (A253), and **(E, F)** the hypopharynx (Detroit 562 and FaDu) was measured by microscopy up to 12 days post-seeding and analysed from three biologically independent experiments.

**Figure 6:**
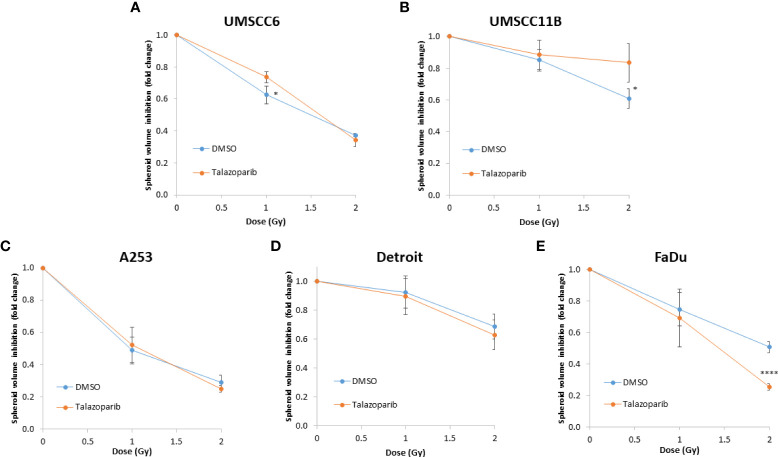
Impact of talazoparib on the enhancement of the radiosensitivity of HPV-negative HNSCC spheroids. **(A–E)** The fold growth of HPV-negative HNSCC spheroids from days 3 to 12 post-seeding was determined relative to the x-ray radiation dose, and this was normalised to the unirradiated control which was set to 1.0. *p < 0.05, ****p < 0.001 as analysed by a two sample t-test.

The effectiveness of PARP inhibition in sensitising cells has been linked to the PARP trapping potency. Therefore, we examined the impact of the strong PARP trapper talazoparib in enhancing the radiosensitivity of HNSCC cells grown as 3D spheroids, focussing on the HPV-negative HNSCC spheroids due to their inherent radioresistance. In terms of OPSCC spheroids, talazoparib alone at the concentration tested (0.1 µM) had a dramatic impact on UMSCC74A spheroids where growth was almost completely supressed (**Figure 5A**, **Supplementary Figure 6**), whereas the growth inhibition (2.2-fold) in UMSCC6 spheroids was comparatively less (**Figure 5B**, **Table 2**, **Supplementary Figure 6**). Talazoparib was able to enhance the radiosensitivity of UMSCC6 spheroids, and where growth was reduced by 1.8-2.4 fold (at 1 and 2 Gy) compared against the respective DMSO-treated spheroids. For the laryngeal spheroid model (UMSCC11B), growth was again significantly reduced by talazoparib only (by 7.7-fold), but there was marked enhancement in radiosensitivity with the combination of talazoparib and x-rays evident by the 6.6-fold (1 Gy) and 5.6-fold (2 Gy) growth inhibition (**Figure 5C**, **Table 2**, **Supplementary Figure 6**). Using spheroids derived from salivary gland cells (A253), growth was inhibited by 1.7-fold by talazoparib alone, but also talazoparib led to increased growth inhibition following irradiation (1.6-fold at 1 Gy and 2.0-fold at 2 Gy) (**Figure 5D**, **Table 2**, **Supplementary Figure 6**). Growth of spheroids derived from HPV-negative cells from the hypopharynx (Detroit 562 and FaDu) was only inhibited by 1.1–1.3-fold in the presence of talazoparib only, whereas this enhanced sensitivity to x-ray radiation (1.2–1.5-fold inhibition at 1 Gy and 1.2–2.7-fold inhibition at 2 Gy) (**Figures 5E, F**, **Table 2**, **Supplementary Figure 6**). However, these observed fold changes in radiosensitivity are relative to the data being compared (e.g., spheroids treated with DMSO and 1 Gy versus inhibitor and 1 Gy) and do not take into account the effect of the inhibitor alone. This is reflected in the analysis of fold decreases in spheroid volume relative to radiation dose to analyse for synergy with PARP inhibition, which revealed only significantly enhanced radiosensitivity of FaDu spheroids by talazoparib, whereas there was no impact on the other HPV-negative spheroids (**Figures 6A–E**). This demonstrates that talazoparib largely acts in an additive manner in enhancing radiosensitivity.

### Olaparib and talazoparib enhance the radiosensitivity of HPV-negative HNSCC spheroids to proton beam therapy

**Figure 7:**
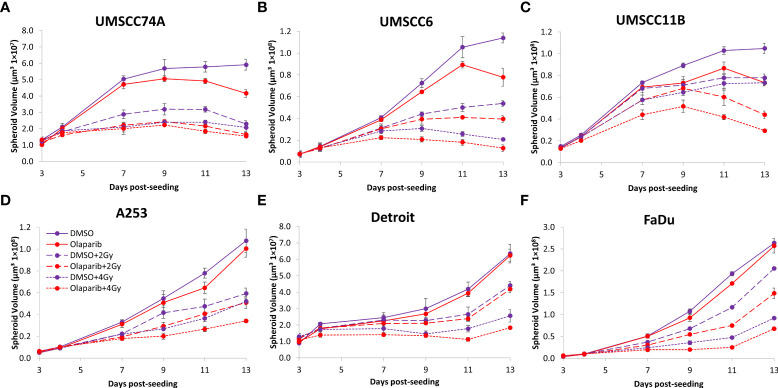
Impact of olaparib on the radiosensitivity and growth of HPV-negative HNSCC spheroids in response to protons. Spheroids were allowed to develop for 24 h in ultra-low-attachment plates, treated with DMSO or olaparib (0.1 µM) for a further 24 h, and then unirradiated or irradiated (2 or 4 Gy) on day 3 with a single dose of protons. Growth of spheroids derived from cells from **(A, B)** the oropharynx (UMSCC74A and UMSCC6), **(C)** the larynx (UMSCC11B), **(D)** the salivary gland (A253), and **(E, F)** the hypopharynx (Detroit 562 and FaDu) was measured by microscopy up to 13 days post-seeding and analysed from three biologically independent experiments.

**Figure 8:**
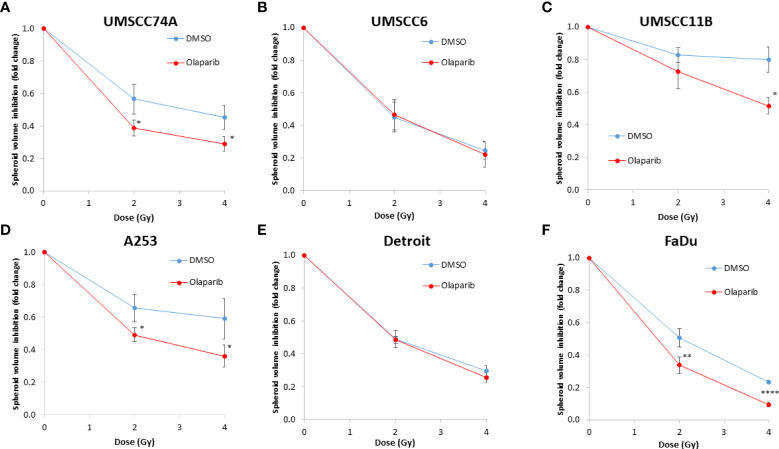
Impact of olaparib on the enhancement of the radiosensitivity of HPV-negative HNSCC spheroids to protons. **(A–F)** The fold growth of HPV-negative HNSCC spheroids from days 3 to 11 post-seeding was determined relative to the proton dose, and this was normalised to the unirradiated control which was set to 1.0. *p < 0.05, **p < 0.02, ****p < 0.001 as analysed by a two-sample t-test.

We extended our observations of the impact of the PARP inhibitors olaparib and talazoparib in radiosensitising HPV-negative HNSCC 3D spheroids by examining the effects in response to proton beam therapy, which is a precision-targeted modality that is increasingly being utilised for the treatment of HNSCC patients (25, 26). In OPSCC spheroids (UMSCC74A and UMSCC6), olaparib in combination with protons was able to supress spheroid growth by 1.2–1.3-fold (at 2 Gy) and 1.3–1.4-fold (at 4 Gy) compared against the respective DMSO-treated spheroids (**Figures 7A, B**, **Table 3**, **Supplementary Figure 7**). In the laryngeal (UMSCC11B) and salivary gland (A253) spheroid models, growth was similarly reduced by 1.3-fold (2 Gy) and 1.6-1.7-fold (4 Gy) following the combination of both olaparib and proton irradiation (**Figures 7C, D**, **Table 3**, **Supplementary Figure 7**). Spheroids derived from HPV-negative cells from the hypopharynx were radiosensitised to different extents in the presence of olaparib. Spheroid growth was inhibited in Detroit 562 models by 1.2-fold (at 2 Gy) and 1.4-fold (at 4 Gy), whereas sensitivity to the combination of olaparib and proton irradiation in the FaDu spheroid models was observed to be higher through a 1.4-fold (at 2 Gy) and 2.4-fold (at 4 Gy) inhibition (**Figures 7E, F**, **Table 3**, **Supplementary Figure 7**). Analysis of fold decreases in spheroid volume relative to proton dose revealed significantly enhanced radiosensitivity of UMSCC74A, UMSCC11B, A253, and FaDu spheroids by olaparib in a synergistic manner (**Figures 8A, C, D, F**), whereas there was no impact on UMSCC6 and Detroit 562 spheroids (**Figure 8B, E**).

**Table 3:** Olaparib and talazoparib selectively enhance the sensitivity of HPV-negative HNSCC spheroids in response to proton irradiation.

Treatment	UMSCC6	UMSCC74A	UMSCC11B	A253	Detroit 562	FaDu
Ola+2 Gy	1.2 ± 0.1	1.3 ± 0.4	1.3 ± 0.2	1.3 ± 0.2	1.2 ± 0.0	1.4 ± 0.1
Ola+4 Gy	1.3 ± 0.2	1.4 ± 0.1	1.7 ± 0.4	1.6 ± 0.1	1.4 ± 0.4	2.4 ± 0.4
Tala+2 Gy	2.6 ± 0.5	n.d.	1.8 ± 0.3	1.4 ± 0.1	2.8 ± 1.1	2.3 ± 0.5
Tala+4 Gy	3.1 ± 0.2	n.d.	2.4 ± 0.6	3.0 ± 0.2	3.6 ± 0.6	3.1 ± 0.5

Growth inhibition ratios (mean ± S.D) comparing the fold increase in spheroid volume between days 3 and 11 following olaparib or talazoparib versus the appropriate DMSO controls (alone, or combination with protons) were calculated in HPV-negative and HPV-positive HNSCC spheroids. n.d. refers to not determined.

**Figure 9:**
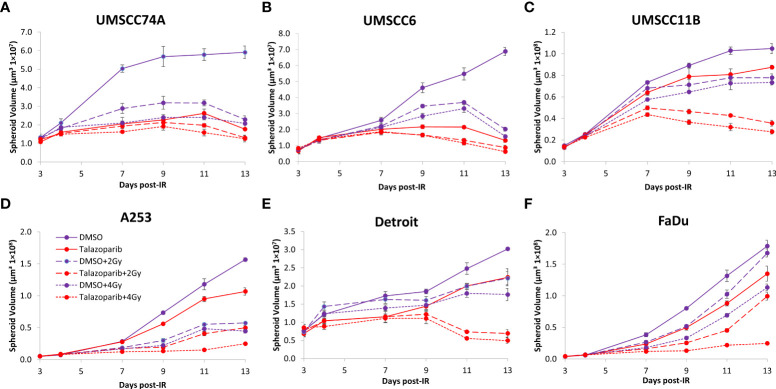
Impact of talazoparib on the radiosensitivity and growth of HPV-negative HNSCC spheroids in response to protons. Spheroids were allowed to develop for 24 h in ultra-low-attachment plates, treated with DMSO or talazoparib (0.1 µM) for a further 24 h, and then unirradiated or irradiated (1 or 2 Gy) on day 3 with a single dose of protons. Growth of spheroids derived from cells from **(A, B)** the oropharynx (UMSCC74A and UMSCC6), **(C)** the larynx (UMSCC11B), **(D)** the salivary gland (A253), and **(E, F)** the hypopharynx (Detroit 562 and FaDu) was measured by microscopy up to 13 days post-seeding and analysed from three biologically independent experiments.

**Figure 10:**
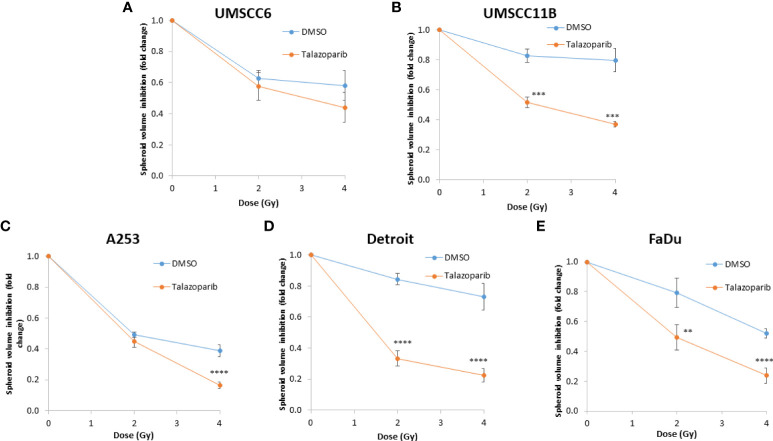
Impact of talazoparib on the enhancement of the radiosensitivity of HPV-negative HNSCC spheroids to protons. **(A–E)** The fold growth of HPV-negative HNSCC spheroids from days 3 to 11 post-seeding was determined relative to the proton dose, and this was normalised to the unirradiated control which was set to 1.0. **p < 0.02, ***p < 0.01, ****p < 0.001 as analysed by a two-sample t-test.

In OPSCC spheroids (UMSCC74A and UMSCC6), talazoparib alone was again notably effective in significantly inhibiting growth of these models. In combination with protons, talazoparib was able to suppress growth of UMSCC6 spheroids by 2.6- and 3.1-fold (at 2 and 4 Gy) compared against the respective DMSO-treated spheroids, therefore working additively in enhancing radiosensitivity (**Figures 9A, B**, **Table 3**, **Supplementary Figure 8**). In the laryngeal (UMSCC11B) spheroids, growth was markedly inhibited by 1.8-fold (2 Gy) and 2.4-fold (4 Gy), and similarly in salivary gland (A253) spheroid models, growth was reduced by 1.4-fold (2 Gy) and 3.0-fold (4 Gy) following the combination of both talazoparib and proton irradiation (**Figures 9C, D**, **Table 3**, **Supplementary Figure 8**). Interestingly, both spheroid models derived from the hypopharynx (FaDu and Detroit 562) displayed markedly enhanced sensitivity to proton irradiation in the presence of talazoparib. Spheroid growth inhibition of 2.8–3.6-fold (FaDu) and 2.3–3.1-fold (Detroit 562) was observed at 2–4 Gy (**Figures 9E, F**, **Table 3**, **Supplementary Figure 8**). These data are supported by analysis of fold decreases in spheroid volume relative to proton dose, which demonstrate enhanced radiosensitivity of the majority of the spheroid models in a synergistic manner, apart from UMSCC74A where talazoparib is a potent inhibitor of spheroid growth alone. Indeed, there was an observed significant radiosensitisation of UMSCC11B, A253, Detroit 562, and FaDu spheroids synergistically by talazoparib (**Figures 10A–E**).

### Enhanced sensitivity of HPV-negative HNSCC spheroids to PARP inhibition appears to correlate with HR deficiency

**Figure 11:**
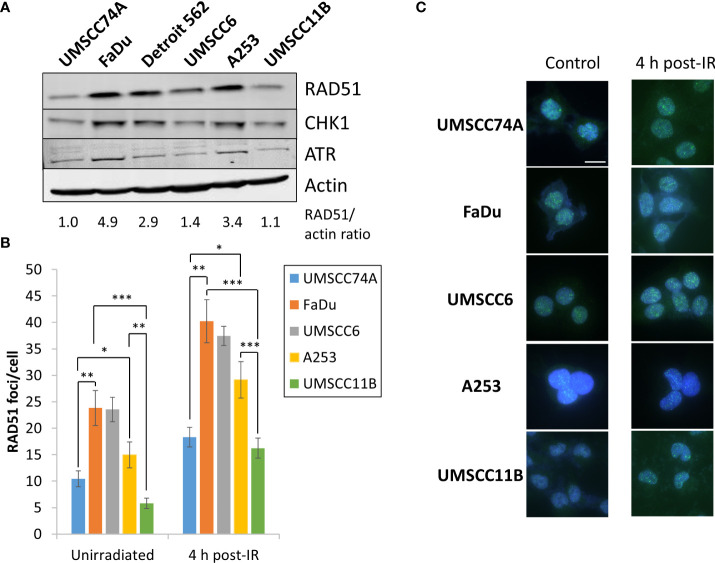
Analysis of the protein levels of HR-related enzymes in HPV-negative HNSCC cells. **(A)** Whole-cell extracts from HPV-negative HNSCC cells were prepared and analysed by immunoblotting with RAD51, CHK1, ATR, or actin antibodies. The ratio of RAD51 relative to actin in the cell extracts, normalised to those in UMSCC74A cells which was set to 1.0, is shown. **(B, C)** RAD51 foci were analysed by immunofluorescent staining in unirradiated HNSCC cells, and at 4 h post-irradiation (4 Gy) with x-rays. **(B)** Shown is the mean number of foci/nucleus with standard deviations from three independent experiments. **(C)** Shown are representative images of RAD51 foci (green) within cell nuclei (blue). *p < 0.05, **p < 0.001, ***p < 0.0001, as analysed by a one-sample t-test.

PARP inhibitors are well established to be effective in the killing of HR-deficient cells and tumours *via* synthetic lethality (16, 17). We therefore predicted that the effectiveness of olaparib and talazoparib, particularly alone but also in combination with IR, in supressing the growth of HPV-negative HNSCC spheroids is linked to their efficiency of HR. Notably, we observed from the above experiments that the growth of UMSCC74A, UMSCC11B, and to some extent UMSCC6 spheroids were sensitive to PARP inhibition alone, whereas FaDu, Detroit 562, and to a lesser extent A253 spheroids were relatively insensitive. Using immunoblotting, we demonstrate that the levels of the key HR protein RAD51 are higher (by 2.9–4.9-fold) in FaDu, Detroit 562, and A253 cells that show PARP inhibitor resistance, compared to UMSCC74A and UMSCC11B cells that are PARP inhibitor sensitive (**Figure 11A**). The protein levels of the signalling enzymes ATR and CHK1 are also relatively higher in these cells (specifically, ATR is 1.4–3.6-fold higher in FaDu and Detroit 562 compared to UMSCC74A and UMSCC11B cells, whereas CHK1 is 1.5–2.9-fold higher in FaDu, Detroit 562, and A253 compared to UMSCC74A and UMSCC11B cells). We also show that the number of RAD51 foci/cell in unirradiated cells, as well as in cells 4 h post-irradiation (with 4 Gy), is significantly higher in FaDu and A253 cells compared to other cells including UMSCC74A and UMSCC11B that show PARP inhibitor sensitivity (**Figures 11B, C**; note that RAD51 foci were not analysed in Detroit 562 due to cell clumping during growth). However surprisingly, UMSCC6 shows a high baseline and IR induced level of RAD51 foci/cell using this assay. Nevertheless, these data indicate that the sensitivity of HNSCC cells to PARP inhibition correlates with key protein levels and efficiency of HR.

## Discussion

It is clear that patients with HPV-positive OPSCC, in comparison to HPV-negative diseases, have an increased response to radiotherapy which leads to an improvement in prognosis and survival rate (5–8). This difference in treatment response has also been observed in cell lines grown as monolayers derived from the respective patients, and furthermore that the increased radiosensitivity of HPV-positive OPSCC has been demonstrated to be as a consequence of defects in the repair of DNA DSBs (9–12). Studies have therefore suggested that PARP inhibitors can be utilised to further radiosensitise HPV-positive OPSCC cells as a consequence of the persistence of DSBs, although data have interestingly also revealed this to be an effective approach in cells from HPV-negative HNSCC even though these are DSB repair proficient (reviewed in (18)). Despite this, there is little preclinical evidence supporting the impact of PARP inhibitors in combination with different radiation modalities (photons and protons), and utilising 3D HNSCC models that more accurately reflect the structure and the treatment of the original tumour. In this study, we have now examined the comparative effect of photons (x-rays) on 3D spheroid models of HPV-positive and HPV-negative OPSCC and also the impact of the PARP inhibitors olaparib and talazoparib in sensitising an extended panel of radioresistant HPV-negative HNSCC models to both photons and proton beam therapy.

We discovered that similar to cells grown as monolayers, growth of two separate 3D spheroid models of HPV-positive OPSCC was more greatly inhibited by x-ray irradiation than two respective HPV-negative OPSCC models, demonstrating their increased radiosensitivity. Despite this, we observed that spheroids derived from HPV-positive OPSCC grew very slowly, reflecting their slow growth also as monolayers, and one of the models (UPCI-SCC154) only grew ~1.6-fold in volume over a 15-day period compared to the others used, limiting its accurate evaluation. We were however able to show using neutral comet assays that the DSB repair capacity of two HPV-positive OPSCC grown as spheroids in response to x-rays was significantly reduced compared to HPV-negative OPSCC. This demonstrates that the HPV-positive OPSCC cells grown as 3D spheroid models still retain inherent deficiencies in DSB repair, which has been observed in a number of studies using monolayer cells utilising both comet assays and analysis of DSB surrogate markers such as γH2AX and 53BP1 *via* immunofluorescence microscopy (9–11).

In addition to observed differences in radiosensitivity based on HPV status, we have shown that the growth of relatively radioresistant OPSCC cells (UMSCC74A and UMSCC6) as 3D spheroids could be inhibited (by 1.3–2.2-fold dependent on the model and dose of x-rays used) in the presence of the PARP inhibitor olaparib. Assessment of the synergy of PARP inhibition with x-ray irradiation, however, revealed that only UMSCC74A was significantly radiosensitised synergistically, whereas in UMSCC6 increased radiosensitisation was largely additive. In comparison, none of the two HPV-positive OPSCC spheroid models showed synergistic radiosensitisation through PARP inhibition. This reflects our previous data using clonogenic assays to measure cell survival post IR in the presence of olaparib, where we observed a greater radiosensitisation of HPV-negative OPSCC (9). In contrast, it has previously been shown that the PARP inhibitor veliparib appears to have a greater effect on radiosensitising the HPV-positive OPSCC cells UMSCC47 and UPCI-SCC154 compared to the HPV-negative UMSCC1 cell line (10). Additionally, three HPV-positive OPSCC cells (UMSCC47, UPCI-SCC154, and UPCI-SC104) appeared to show higher radiosensitisation to veliparib compared to three HPV-negative HNSCC cells (SQD9, SC263, and CAL27) (27). It should be noted though that these studies utilised veliparib, which has a weaker PARP trapper than olaparib or talazoparib. Also, the HPV-negative cell lines used were from different tumour origins (salivary gland and larynx) rather than the specific and comparative oropharyngeal cells used at this point in our study which may explain the discrepancies. To this effect, we observed that HPV-negative HNSCC cells from the larynx, salivary gland, and hypopharynx displayed differential radiosensitisation with x-rays in the presence of olaparib, suggesting tumour cell line variability in the response to the combination treatment. For example, spheroids from UMSCC11B (larynx) were radiosensitised in the presence of olaparib, in a synergistic manner, whereas FaDu and Detroit 562 (hypopharynx) were relatively insensitive to the combination treatment. In fact, these less responsive spheroid models to radiosensitisation through PARP inhibition were found to contain comparatively lower PARP-1 protein levels, but more importantly we discovered increased protein levels and foci of the key HR factor RAD51 compared to the other cells analysed. The variability in response is supported by another study in HPV-negative HNSCC cells (28) and which similarly proposed that the impact of PARP inhibition on radiosensitisation is dependent on the HR proficiency of the cells. Interestingly, downregulation of the receptor tyrosine kinase AXL has been suggested to enhance the response of HNSCC cells (584 and 1386-LN), as well as breast and lung cancer cells, to olaparib and which was linked with reduced levels of RAD51 foci and decreased HR efficiency (29). However, the impact of PARP inhibition in combination with ionising radiation was not investigated. Additionally, the effectiveness of PARP inhibition in the radiosensitisation of HNSCC cells and tumours has been linked with SMAD4 involved in TGFβ signalling and where SMAD4-deficient models were shown to be more responsive to the combined treatment (30). Interestingly and on TCGA analysis, this study also found a correlation between decreased *smad4* and lower *fanc/brca* gene expression suggestive of a **“**BRCAness**”** phenotype. Collectively though, this further demonstrates that more detailed mechanisms of action studies need to be performed to fully understand the key driving factors leading to enhanced radiosensitisation of HNSCC cells through PARP inhibition.

Focussing on relatively radioresistant HPV-negative HNSCC spheroid models from different tumour origins, we analysed the comparative radiosensitisation properties of olaparib and talazoparib, the latter of which is characterised as a strong PARP trapper (21, 22). Whilst we found that talazoparib alone was generally more effective in preventing 3D spheroid growth, and particularly toxic to HPV-negative OPSCC spheroids (UMSCC74A and UMSCC6), we found no overall strong evidence that this led to significantly enhanced radiosensitisation of all HPV-negative HNSCC spheroid models in response to x-ray irradiation in a synergistic manner. This would indicate that PARP trapping is not a critical factor in driving enhanced radiosensitivity of HNSCC models and that inhibition of poly(ADP-ribosyl)ation activity itself (in addition to HR proficiency of the cells) is likely the major determinant through which impact on spheroid growth is achieved in combination with x-ray irradiation. Interestingly, there appeared to be greater differences with the effectiveness of olaparib versus talazoparib in response to proton irradiation. Here we observed that talazoparib in combination with protons led to a more profound synergistic inhibition of growth of HPV-negative HNSCC spheroids than that achieved with olaparib, particularly of those derived from the hypopharynx (FaDu and Detroit 562). The reason behind this difference is currently unclear but could possibly relate to the changes in DNA damage profile or cellular response to the different radiation modalities (31). To this effect, we have recently shown, using similar cell lines employed in this study, that these display some degree of variability in terms of both clonogenic survival and 3D spheroid growth following photon versus proton irradiation, and similarly, differential responses to inhibitors against the DSB repair proteins ATM, ATR, and DNA-Pk also exist (32). We have also shown in this study that there is increased expression of HR factors (RAD51, ATR, and CHK1) in cells resistant to the combination of olaparib and IR (photons and protons). Furthermore, we have shown that monolayer cells, albeit irradiated at the distal end of the Bragg peak with relatively highly linear energy transfer protons, generate complex DNA damage that has a strong dependence on the involvement of PARP-1 for their repair (23, 24). Cumulatively, these studies would suggest that the DNA damage profile and efficiency of the cellular DDR mediated by the DSB repair pathways NHEJ and HR, but also the reliance on one of these pathways, may be responsible for the difference in effectiveness of talazoparib versus olaparib in combination with protons in the current study. However, it is possible that this could also be mediated through differences in metabolism and cell death activation which PARP proteins also critically play a role in (33), but which nevertheless requires further investigation. In addition to this, our ongoing experiments aim to examine the impact of PARP inhibition both alone, but particularly on the radiosensitisation of patient-derived HNSCC organoids, with a view to providing more preclinical evidence that this is a strategy that could be taken forward for future benefit of HNSCC patients.

## Data availability statement

The raw data supporting the conclusions of this article will be made available by the authors, without undue reservation.

## Author contributions

JP conceptualized and designed the project. CZ and JP designed the experimental setup. CZ, MF, and JH performed experiments. CZ, MF, JH, GG, and JP performed data analysis and validation. CZ and JP wrote the manuscript, and all authors contributed to reviewing and editing. JP coordinated funding acquisition. All authors contributed to the article and approved the submitted version.

## Funding

This research was supported by funding from North West Cancer Research (CR1197).

## Acknowledgments

The authors thank Prof T. Carey and Dr S. Gollin for providing HNSCC cells. We also thank Linda Mortimer and the technical team at the Clatterbridge Cancer Centre for assistance with proton irradiation of cells.

## Conflict of interest

The authors declare that the research was conducted in the absence of any commercial or financial relationships that could be construed as a potential conflict of interest.

## Publisher’s note

All claims expressed in this article are solely those of the authors and do not necessarily represent those of their affiliated organizations, or those of the publisher, the editors and the reviewers. Any product that may be evaluated in this article, or claim that may be made by its manufacturer, is not guaranteed or endorsed by the publisher.
