# Supplementary material for: Effectiveness of PARP inhibition in enhancing the radiosensitivity of 3D spheroids of head and neck squamous cell carcinoma
Source: Front Oncol. 2022 Aug 16;12:940377. doi: 10.3389/fonc.2022.940377 (PMC9424551; doi:10.3389/fonc.2022.940377)
Supplement: Supplementary file 1 [file DataSheet_1.docx]

Supplementary Material

**
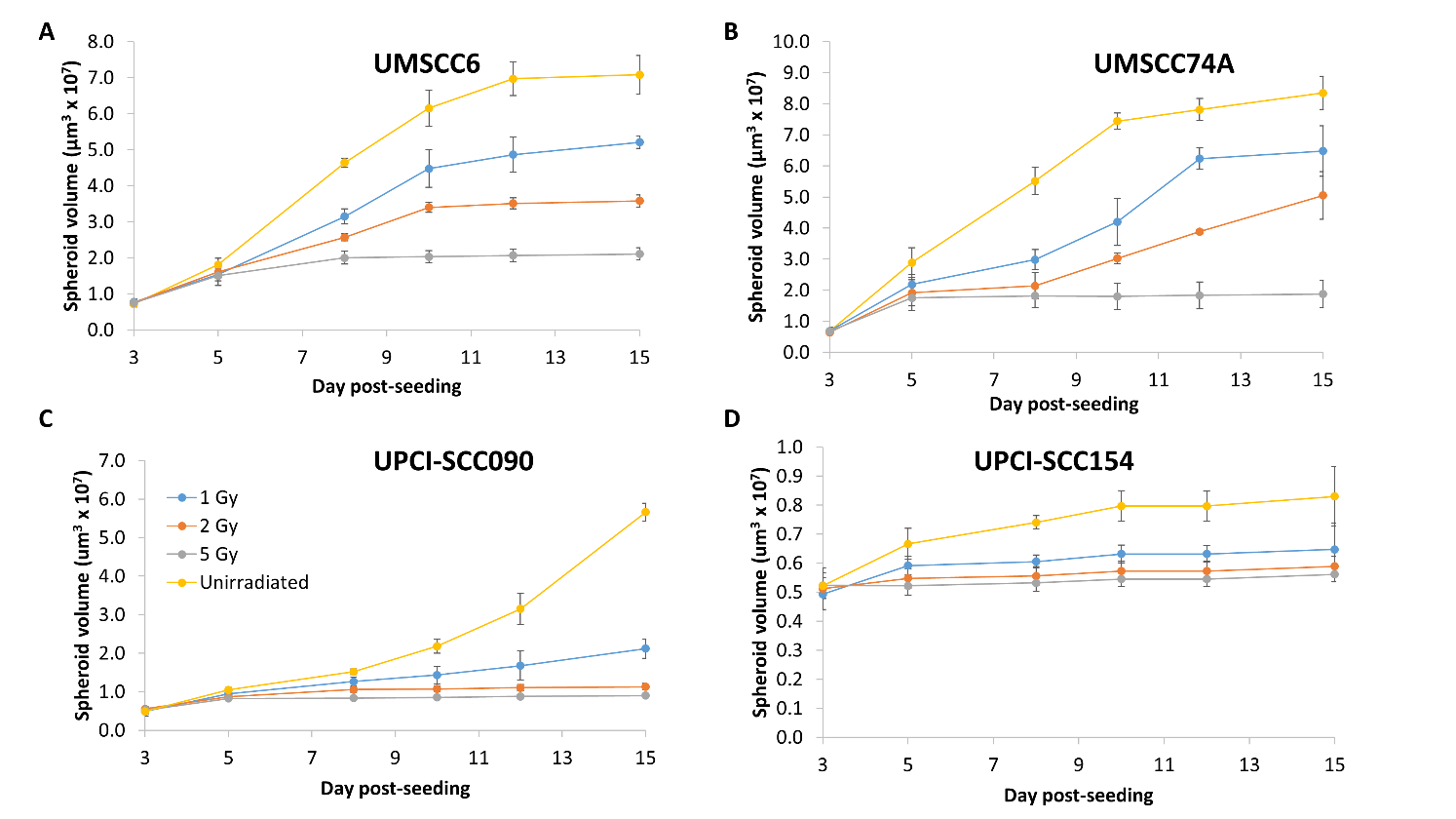
**

**Supplementary Figure 1.** Comparative growth and radiosensitivity of HPV-positive and HPV-negative OPSCC spheroids. Spheroids were allowed to develop for 48 h in ultra-low attachment plates, and then unirradiated or irradiated (1, 2 or 5 Gy) on day 3 with a single dose of x-rays. Growth of (**A**,**B**) HPV-negative OPSCC spheroids (UMSCC6 and UMSCC74A) and (**C**,**D**) HPV-positive OPSCC spheroids (UPCI-SCC090 and UPCI-SCC154) was measured by microscopy up to 15 days post-seeding and analysed from three biologically independent experiments.


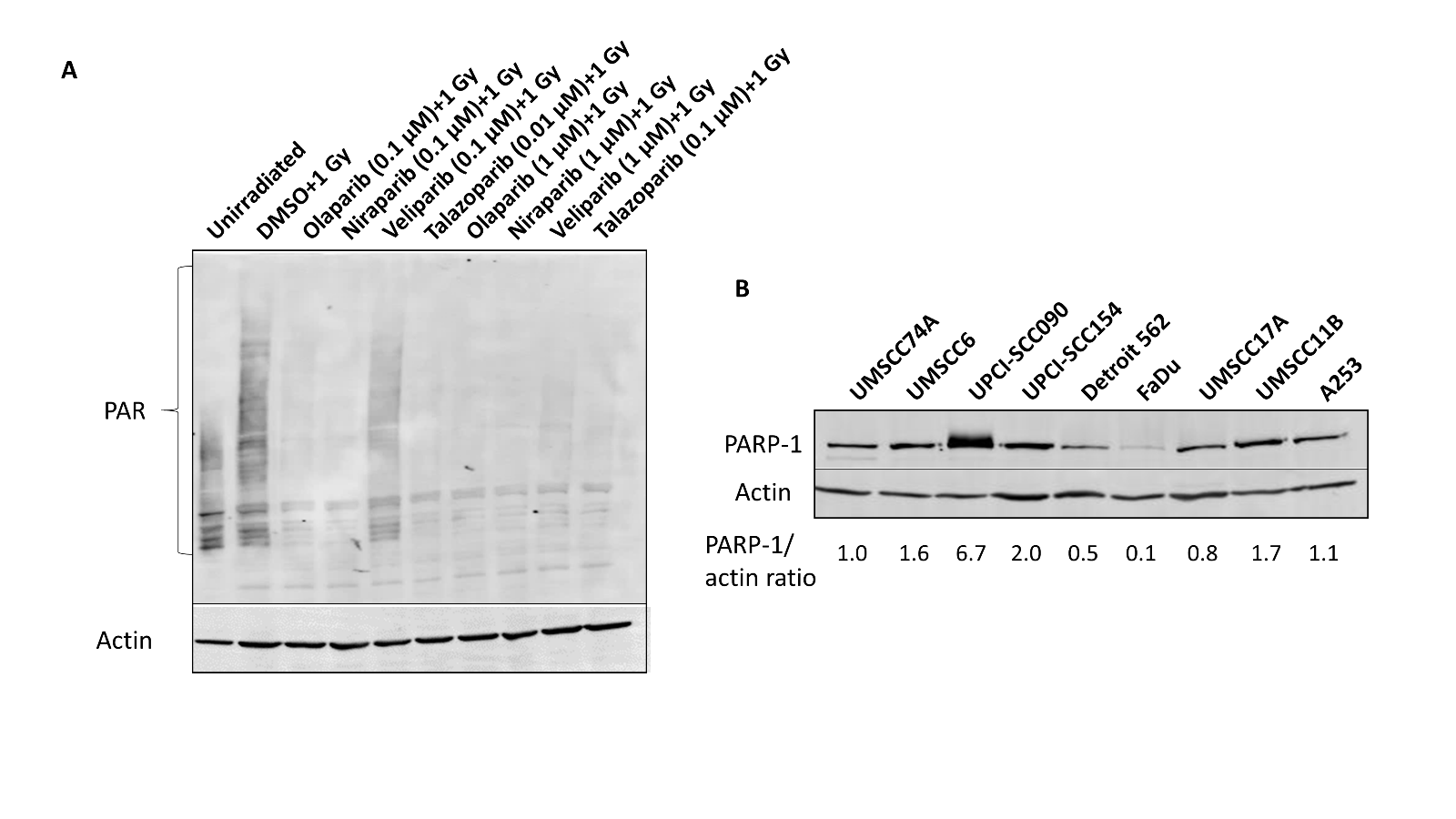


**Supplementary Figure 2.** Inhibition of IR-induced poly(ADP-ribosyl)ation in HPV-negative OPSCC cells and analysis of PARP-1 protein levels in HPV-negative and HPV-positive HNSCC cells. HPV-negative (UMSCC74A) OPSCC cells were preincubated with the PARP inhibitors olaparib, niraparib, veliparib or talazoparib at the indicated concentrations for 24 h, and then either unirradiated or irradiated with 4 Gy x-rays and harvested at 15 min post-irradiation. (A) Whole cell extracts were prepared and analysed by immunoblotting with either poly(ADP-ribose) (PAR) polymers or actin antibodies. (B) Whole cell extracts were prepared from the multiple HPV-negative and HPV-positive HNSCC cells used in this study, and analysed by immunoblotting with either poly(ADP-ribose) polymerase-1 (PARP-1) or actin antibodies. Representative images are shown.


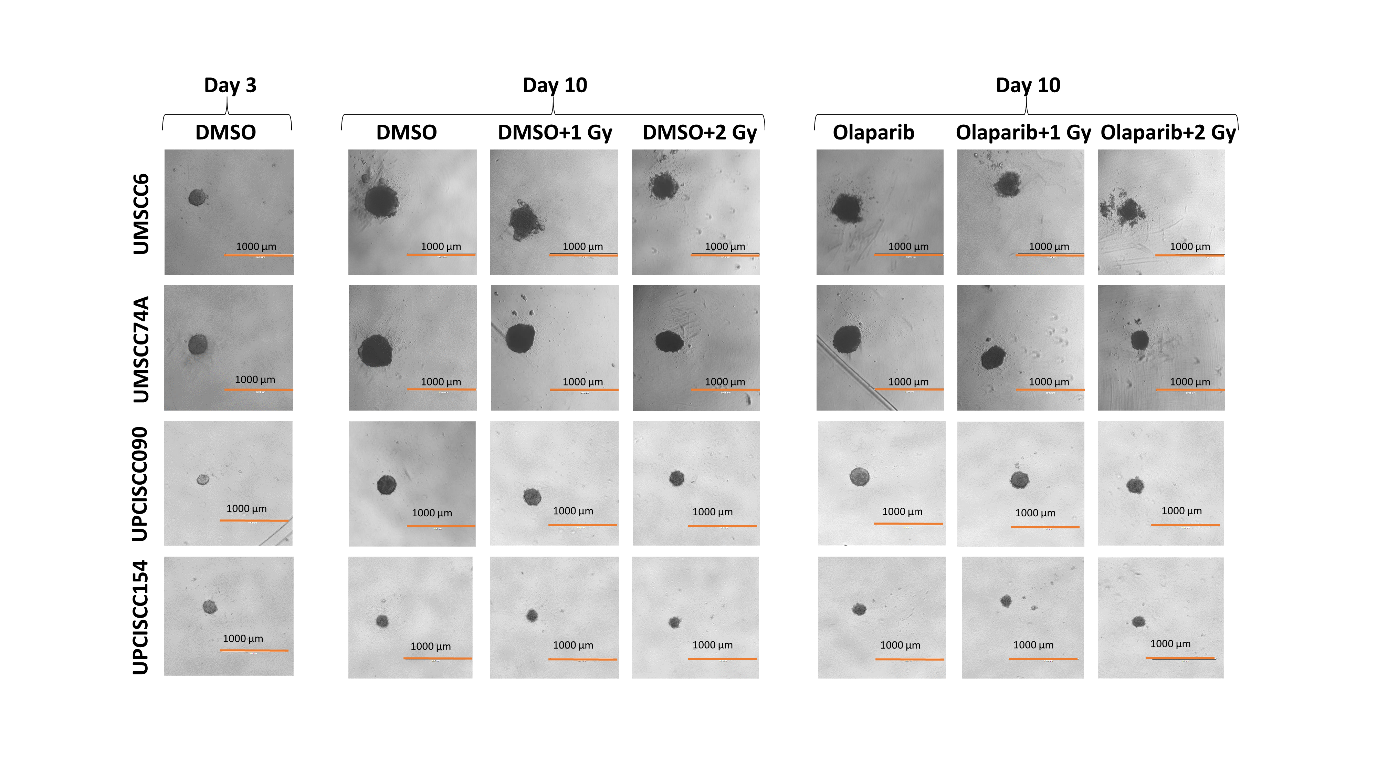


**Supplementary Figure 3.** Inhibition of HPV-negative and HPV-positive OPSCC spheroid growth by olaparib in combination with x-ray irradiation. HPV-negative (UMSCC6 and UMSCC74A) and HPV-positive (UPCI-SCC090 and UPCI-SCC154) OPSCC cells were plated into 96-well ultra-low attachment plates and spheroids allowed to form for 48 h (Day 3). Spheroids were pretreated with either DMSO (as a vehicle only control) or olaparib (0.1 µM) for 24 h, and then irradiated with a single 1 Gy or 2 Gy dose of x-rays. Spheroid growth was analysed by microscopy and shown are the respective images at Day 3 and 10 post-seeding.


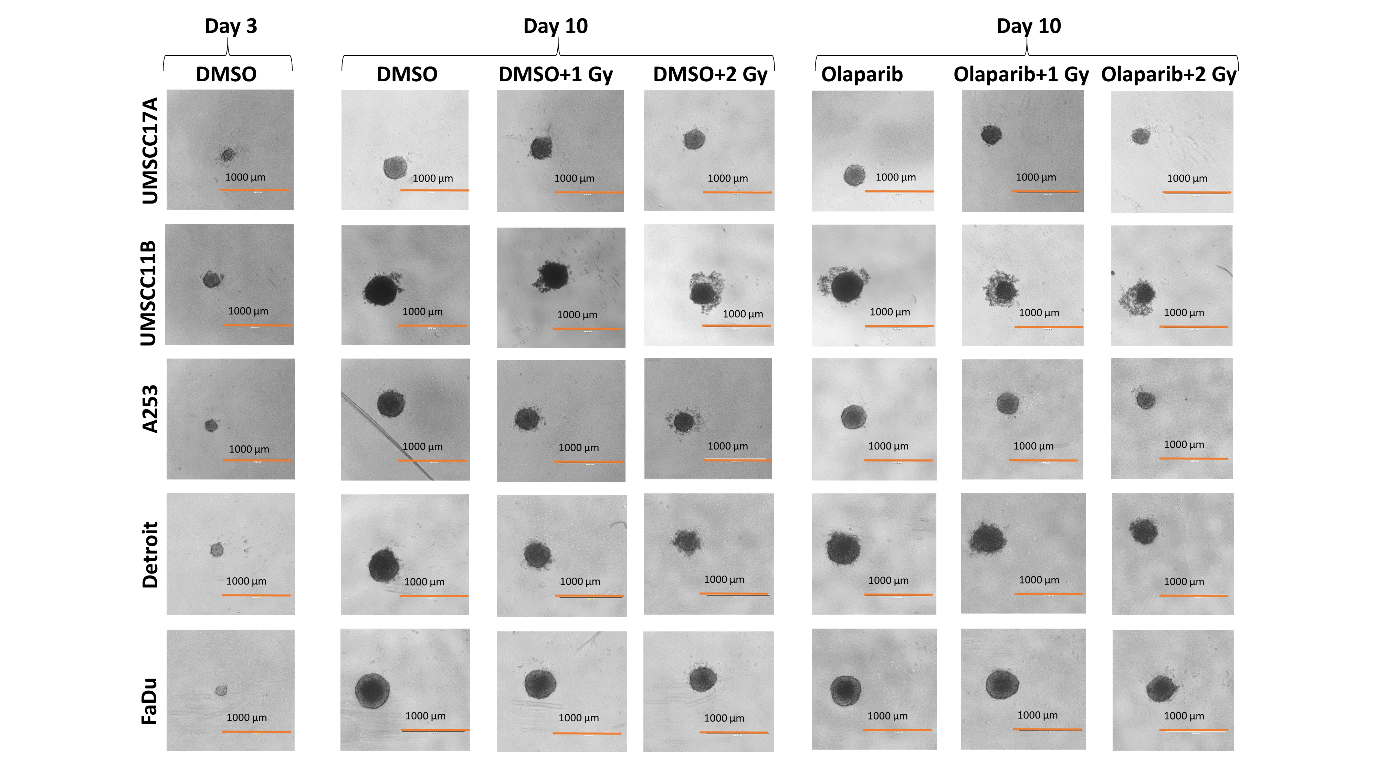


**Supplementary Figure 4.** Inhibition of HPV-negative HNSCC spheroid growth by olaparib in combination with x-ray irradiation. HPV-negative (UMSCC17A, UMSCC11B, A253, Detroit 562 and FaDu) HNSCC cells were plated into 96-well ultra-low attachment plates and spheroids allowed to form for 48 h (Day 3). Spheroids were pretreated with either DMSO (as a vehicle only control) or olaparib (0.1 µM) for 24 h, and then irradiated with a single 1 Gy or 2 Gy dose of x-rays. Spheroid growth was analysed by microscopy and shown are the respective images at Day 3 and 10 post-seeding.

**
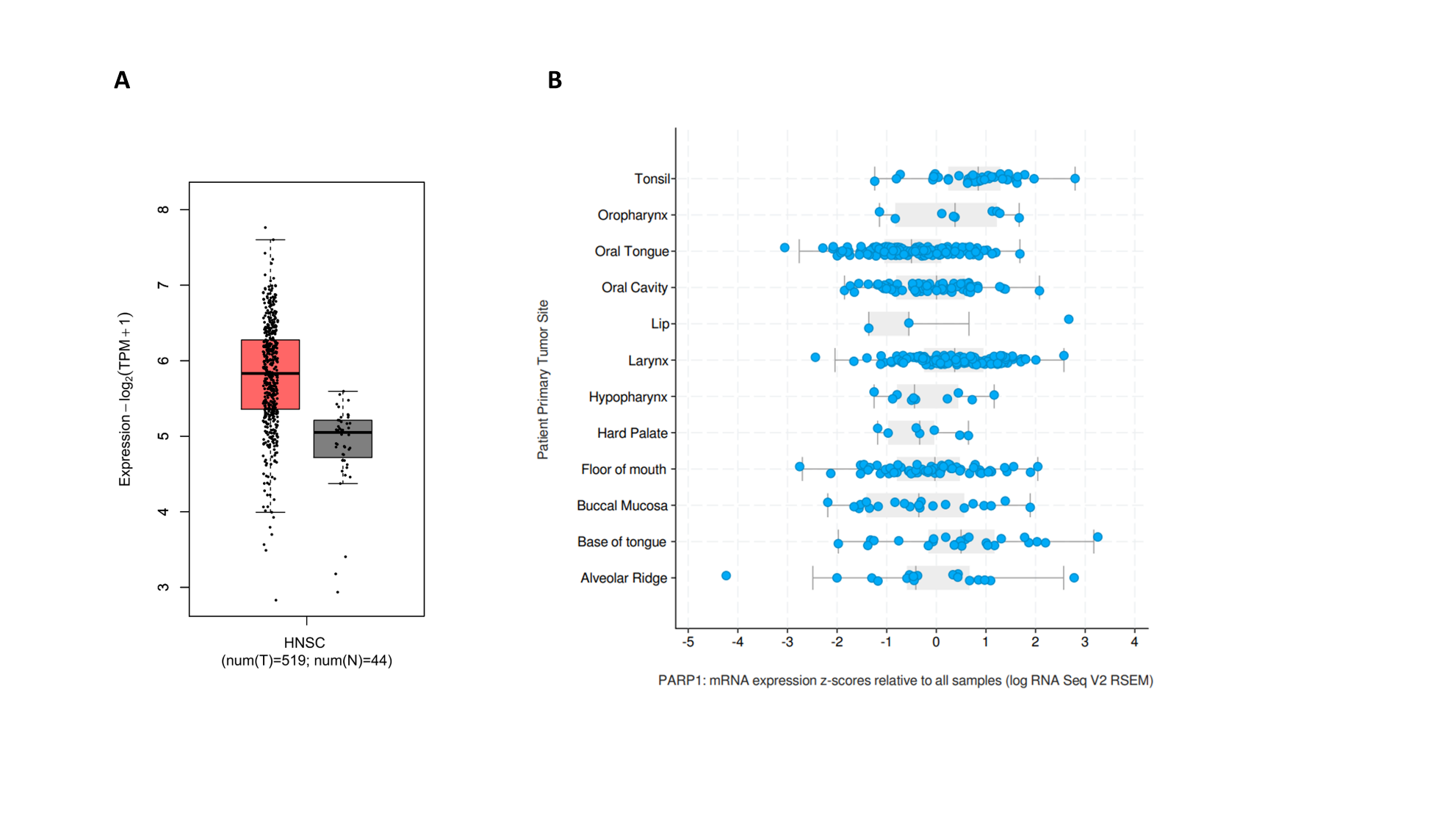
**

**Supplementary Figure 5.** Expression of *parp-1* mRNA in HNSCC tumours. Analysis of *parp-1* mRNA gene expression from the TCGA database in (**A**) 519 HNSCC tumours (red) compared to 44 paired normal tissues (grey), and (**B**) in different HNSCC primary tumour sites, including the oropharynx, larynx and hypopharynx. Data is displayed using gepia2.cancer-pku.cn and cbioportal.org, respectively.


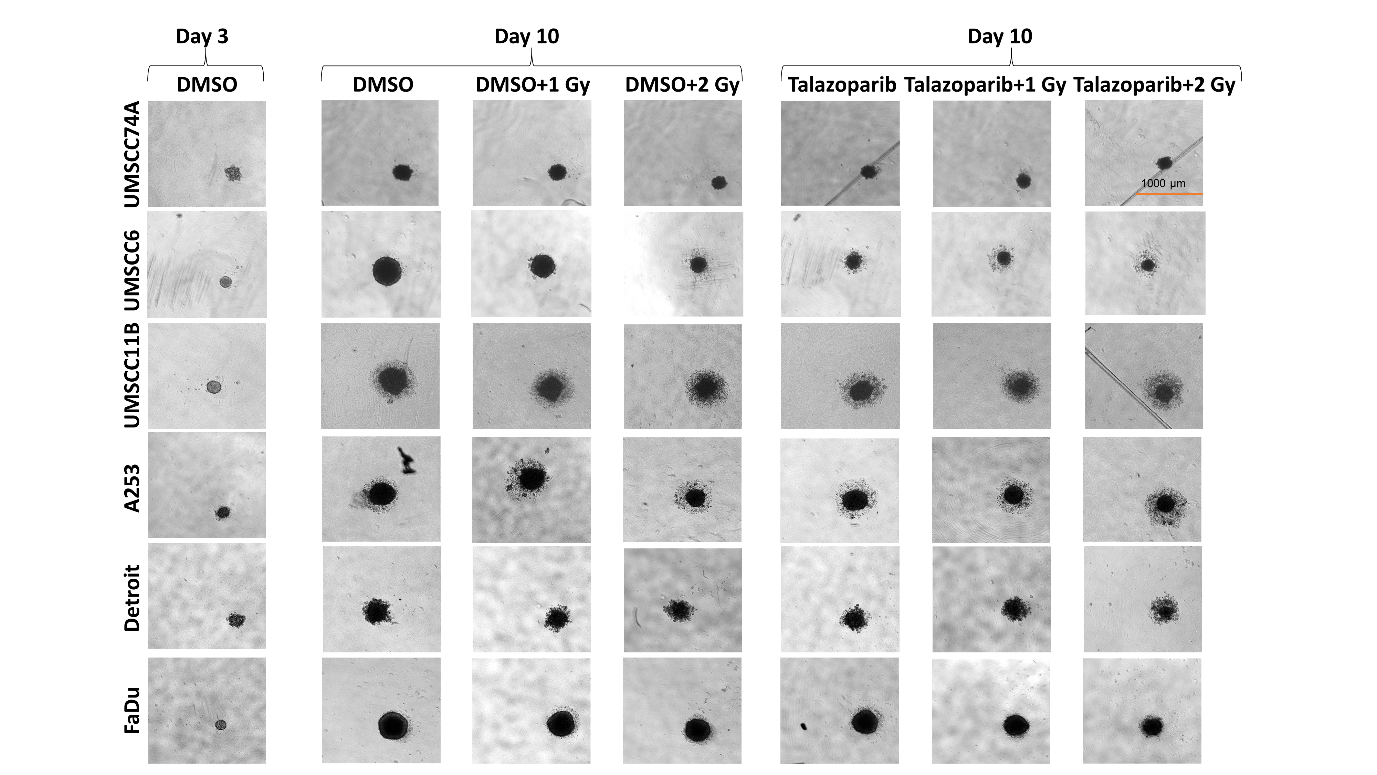


**Supplementary Figure 6.** Inhibition of HPV-negative HNSCC spheroid growth by talazoparib in combination with x-ray irradiation. HPV-negative (UMSCC74A, UMSCC6, UMSCC11B, A253, Detroit 562 and FaDu) HNSCC cells were plated into 96-well ultra-low attachment plates and spheroids allowed to form for 48 h (Day 3). Spheroids were pretreated with either DMSO (as a vehicle only control) or talazoparib (0.1 µM) for 24 h, and then irradiated with a single 1 Gy or 2 Gy dose of x-rays. Spheroid growth was analysed by microscopy and shown are the respective images at Day 3 and 10 post-seeding.


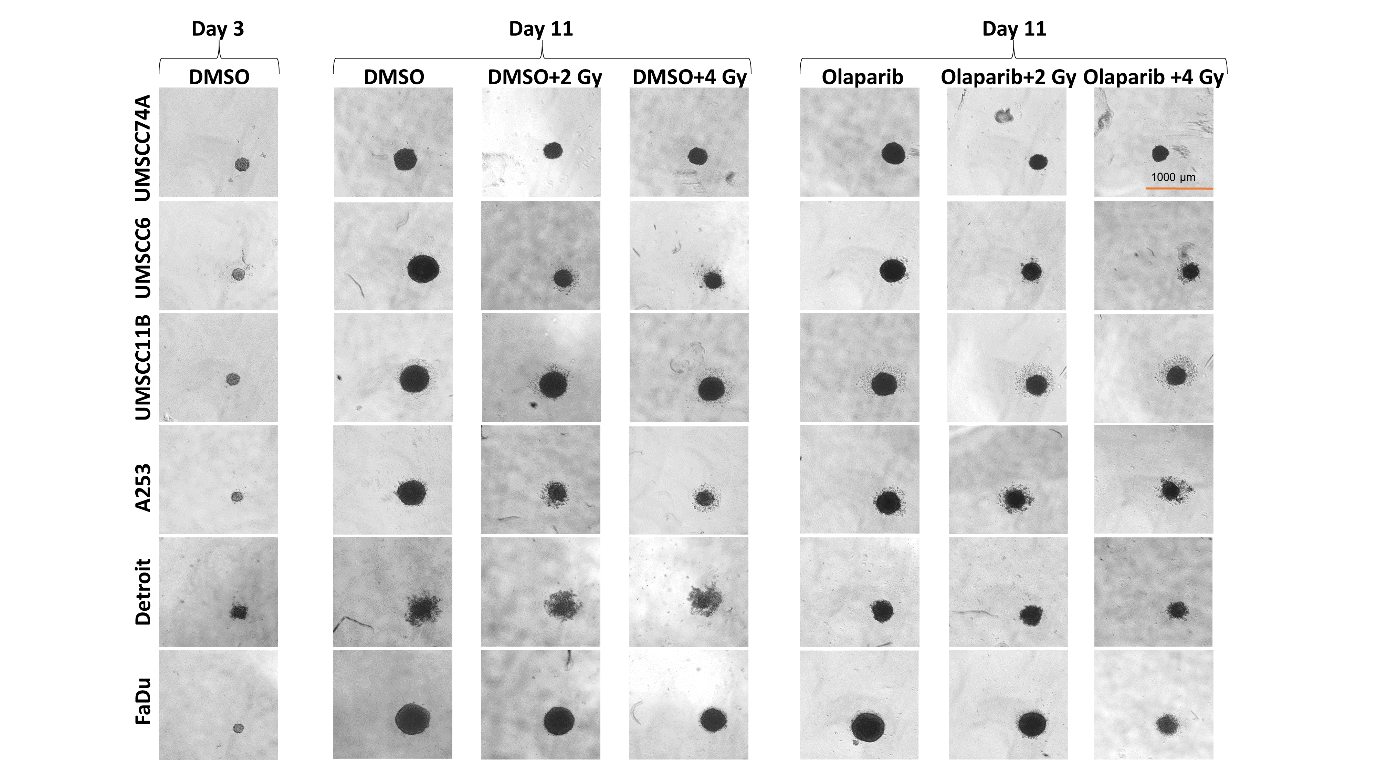


**Supplementary Figure 7.** Inhibition of HPV-negative HNSCC spheroid growth by olaparib in combination with proton irradiation. HPV-negative (UMSCC74A, UMSCC6, UMSCC11B, A253, Detroit 562 and FaDu) HNSCC cells were plated into 96-well ultra-low attachment plates and spheroids allowed to form for 48 h (Day 3). Spheroids were pretreated with either DMSO (as a vehicle only control) or olaparib (0.1 µM) for 24 h, and then irradiated with a single 1 Gy or 2 Gy dose of protons. Spheroid growth was analysed by microscopy and shown are the respective images at Day 3 and 11 post-seeding.


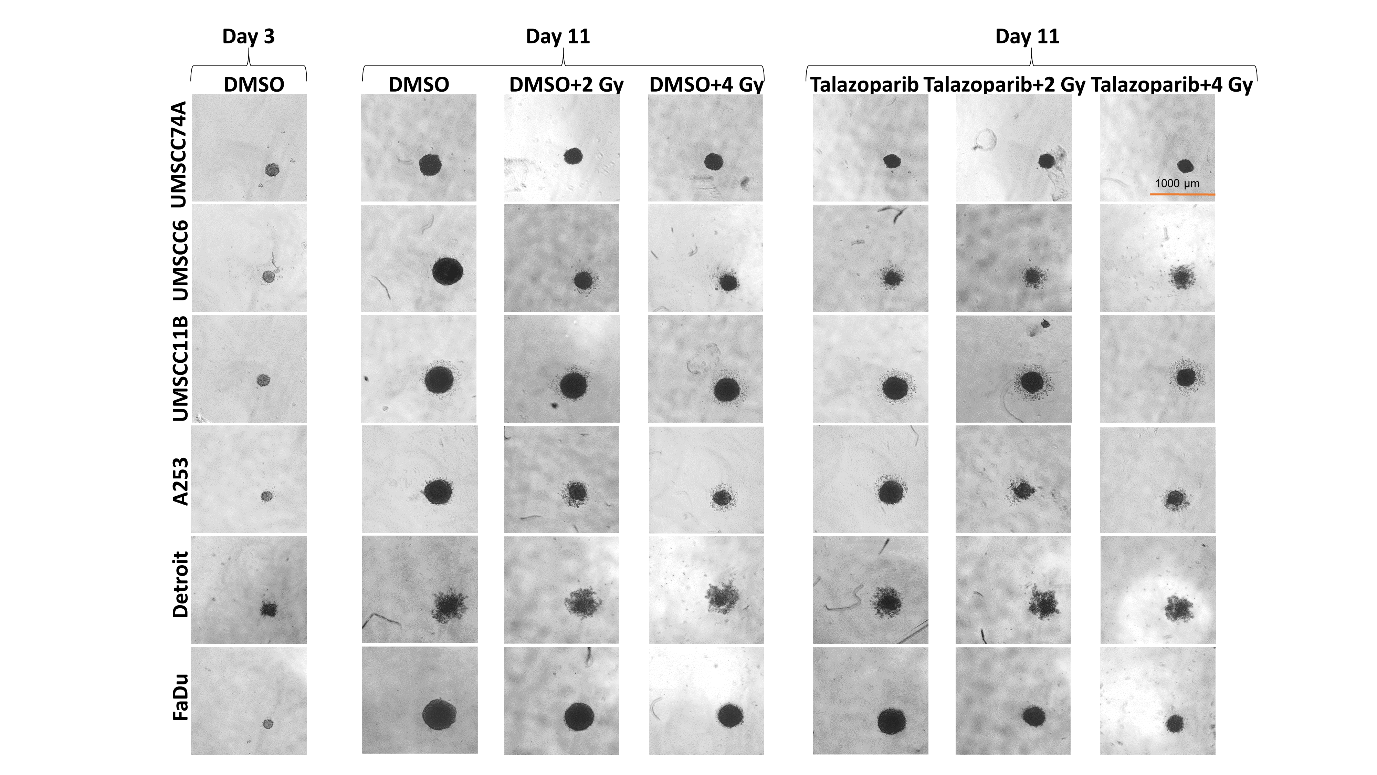


**Supplementary Figure 8.** Inhibition of HPV-negative HNSCC spheroid growth by talazoparib in combination with proton irradiation. HPV-negative (UMSCC74A, UMSCC6, UMSCC11B, A253, Detroit 562 and FaDu) HNSCC cells were plated into 96-well ultra-low attachment plates and spheroids allowed to form for 48 h (Day 3). Spheroids were pretreated with either DMSO (as a vehicle only control) or talazoparib (0.1 µM) for 24 h, and then irradiated with a single 1 Gy or 2 Gy dose of protons. Spheroid growth was analysed by microscopy and shown are the respective images at Day 3 and 11 post-seeding.
